# Influence of *ARHGEF3* and *RHOA* Knockdown on *ACTA2* and Other Genes in Osteoblasts and Osteoclasts

**DOI:** 10.1371/journal.pone.0098116

**Published:** 2014-05-19

**Authors:** Benjamin H. Mullin, Cyril Mamotte, Richard L. Prince, Scott G. Wilson

**Affiliations:** 1 Dept. of Endocrinology and Diabetes, Sir Charles Gairdner Hospital, Nedlands, Western Australia, Australia; 2 School of Biomedical Sciences and CHIRI Biosciences Research Precinct, Faculty of Health Sciences, Curtin University, Bentley, Western Australia, Australia; 3 School of Medicine and Pharmacology, The University of Western Australia, Nedlands, Western Australia, Australia; 4 Twin and Genetic Epidemiology Research Unit, St Thomas’ Hospital Campus, King’s College London, London, United Kingdom; University of Texas Southwestern Medical Center, United States of America

## Abstract

Osteoporosis is a common bone disease that has a strong genetic component. Genome-wide linkage studies have identified the chromosomal region 3p14-p22 as a quantitative trait locus for bone mineral density (BMD). We have previously identified associations between variation in two related genes located in 3p14-p22, *ARHGEF3* and *RHOA*, and BMD in women. In this study we performed knockdown of these genes using small interfering RNA (siRNA) in human osteoblast-like and osteoclast-like cells in culture, with subsequent microarray analysis to identify genes differentially regulated from a list of 264 candidate genes. Validation of selected findings was then carried out in additional human cell lines/cultures using quantitative real-time PCR (qRT-PCR). The qRT-PCR results showed significant down-regulation of the *ACTA2* gene, encoding the cytoskeletal protein alpha 2 actin, in response to *RHOA* knockdown in both osteoblast-like (*P<0.001*) and osteoclast-like cells (*P = 0.002*). *RHOA* knockdown also caused up-regulation of the *PTH1R* gene, encoding the parathyroid hormone 1 receptor, in Saos-2 osteoblast-like cells (*P<0.001*). Other findings included down-regulation of the *TNFRSF11B* gene, encoding osteoprotegerin, in response to *ARHGEF3* knockdown in the Saos-2 and hFOB 1.19 osteoblast-like cells (*P = 0.003–0.02*), and down-regulation of *ARHGDIA*, encoding the Rho GDP dissociation inhibitor alpha, in response to *RHOA* knockdown in osteoclast-like cells (*P<0.001*). These studies identify *ARHGEF3* and *RHOA* as potential regulators of a number of genes in bone cells, including *TNFRSF11B*, *ARHGDIA*, *PTH1R* and *ACTA2*, with influences on the latter evident in both osteoblast-like and osteoclast-like cells. This adds further evidence to previous studies suggesting a role for the *ARHGEF3* and *RHOA* genes in bone metabolism.

## Introduction

Osteoporosis is a common and debilitating bone disease that is characterised by a low bone mineral density (BMD), which leads to an increased risk of fracture [1]. The disease is particularly prevalent in postmenopausal women due to a reduction in oestrogen production, with subsequent effects on bone as well as intestinal and renal calcium handling [2]. In addition to the effects of oestrogen, calcium and other environmental factors on bone structure, there is a strong genetic effect on peak bone mass (attained in early adult life), bone loss and fracture rates [3], [4]. Twin and family studies suggest that 50–90% of the variation in peak bone mass [5]–[7] and 25–68% of the variance in osteoporotic fracture is heritable [4], [8], [9]. The genome-wide linkage scanning approach has identified at least 11 replicated quantitative trait loci (QTL) for BMD [10]–[12], including the 3p14-p22 region of the human genome (LOD 1.1–3.5) [11]–[14].

We have previously identified significant associations between variation in the *RHOA* and *ARHGEF3* genes, which are both located within the 3p14-p22 region, and BMD in women [15], [16]. The functions of these genes are related, with the product of the *ARHGEF3* gene (the Rho guanine nucleotide exchange factor (GEF) 3) specifically activating two members of the RhoGTPase family: RhoA (encoded for by the *RHOA* gene) and RhoB [17]. RhoA is involved with regulating cytoskeletal dynamics and actin polymerisation [18] and has been shown to have a role in osteoblast differentiation [19], [20] and osteoclastic bone resorption [21].

Given the associations that we have previously identified between the *RHOA* and *ARHGEF3* genes and BMD, coupled with the evidence in the literature suggesting a role for RhoA in osteoblasts and osteoclasts, we decided to further investigate the role of these genes in these particular cell types. Knockdown of the *RHOA* and *ARHGEF3* genes was achieved using small interfering RNA (siRNA) in a human osteoblast-like cell line and in osteoclast-like cells derived from a donor, with subsequent microarray analysis to identify genes that were differentially regulated. Replication of selected significant findings was then conducted in additional human osteoblast-like cell lines and in osteoclast-like cells from additional donors.

## Materials and Methods

### Ethics Statement

All subjects that donated blood samples for isolation of peripheral blood mononuclear cells (PBMCs) provided written informed consent and the institutional ethics committee of Curtin University approved the experimental protocol.

### Experimental Approach

To identify genes involved in osteoblast and osteoclast function that are potentially influenced by the *RHOA* and *ARHGEF3* genes, we examined the influence of knockdown of these two genes on 264 candidate genes in an osteoblast-like cell line and osteoclast-like cells obtained from a donor, in triplicate, by microarray analysis. The microarray results showed significant alterations in the expression of a number of the candidate genes, 7 of which were studied in greater detail to validate the findings, based on quantitative real-time PCR (qRT-PCR) studies of the 7 genes in 3 additional osteoblast-like and osteoclast-like cell cultures/lines.

### Cell Culture

The osteoblast-like cell lines used for the gene knockdown experiments included: Saos-2, derived from osteosarcoma tissue (American Type Culture Collection (ATCC) N° HTB-85) [22]; hFOB 1.19, derived from immortalised foetal osteoblasts (ATCC N° CRL-11372) [23]; and MG-63, derived from osteosarcoma tissue (ATCC N° CRL-1427) [24]. These cell lines are all human in origin and were cultured in DMEM (Sigma-Aldrich, St. Louis, USA) pH 7.4 supplemented with 4.77 g/l HEPES, 3.7 g/l NaHCO_3_, 10% (v/v) foetal bovine serum (FBS) and 1% (v/v) penicillin/streptomycin (100 units penicillin and 100 µg streptomycin per ml of media). The osteoclast-like cells used in these studies were differentiated from PBMCs (process described below) and were cultured in α-MEM (Invitrogen, Carlsbad, USA) pH 7.4 supplemented with 2.2 g/l NaHCO_3_, 10% (v/v) FBS and 1% (v/v) penicillin/streptomycin. All cells were cultured at 37°C with 5% CO_2_ and the medium was changed every 2–3 days. Total RNA was harvested from each culture using the RNeasy Mini Kit (Qiagen, Hilden, Germany) and reverse transcription of the RNA was performed using the QuantiTect Reverse Transcription Kit (Qiagen, Hilden, Germany). Quantitation of total RNA was performed using an ND-1000 spectrophotometer (NanoDrop Technologies, Wilmington, USA).

### Isolation of Peripheral Blood Mononuclear Cells and Osteoclastogenesis

Osteoclast-like cells were differentiated from PBMCs isolated from 4 male donors of European descent aged 48±15 years (mean ± SD). Each batch of cells was isolated from 30 ml whole blood collected in 10 ml K_2_EDTA Vacutainer tubes (Becton, Dickinson and Company, Franklin Lakes, USA). Anti-coagulated whole blood samples were centrifuged at 2,200 rpm for 10 min at room temperature before buffy coats were collected and diluted to a total volume of 4 ml with 1× phosphate buffered saline (PBS). The cell suspension was then gently layered over 3 ml of Ficoll-Paque (Pfizer, New York, USA) before being centrifuged again at 1,600 rpm for 40 min at room temperature. The PBMC layer was collected and washed by re-suspension in 6 ml 1× PBS and centrifuged at 800 rpm for 10 min at room temperature. The wash step was repeated on the cell pellet before the cells were re-suspended in 5 ml medium supplemented with 10 ng/ml macrophage colony stimulating factor (M-CSF) (Invitrogen, Carlsbad, USA) and seeded directly into either a 24-well tissue culture plate or 25 cm^2^ tissue culture flask. After two days, the medium was replaced with medium supplemented with 10 ng/ml M-CSF and 100 ng/ml receptor activator of nuclear factor kappa-B ligand (RANKL) (Invitrogen, Carlsbad, USA). The cells were then grown using this medium formulation for 17 days while osteoclastogenesis occurred.

Osteoclast-like cells were stained for tartrate resistant acid phosphatase (TRAP) using a chromogenic TRAP enzyme substrate to confirm production of the TRAP enzyme as an indicator of the osteoclast phenotype. This involved washing the cells with 1× PBS, fixation with 4% (v/v) paraformaldehyde for 15 min, washing 3 times with 1× PBS before incubation with filtered TRAP stain solution at 37°C for 25 min. The stained cells were then washed 3 times with 1× PBS prior to visualisation using light microscopy.

### siRNA Knockdown

Transfection of cells with siRNA sequences was used to knockdown expression of the *ARHGEF3* and *RHOA* genes. Transfections were performed using HiPerFect Transfection Reagent (Qiagen, Hilden, Germany). Two different siRNA sequences were used in tandem to knockdown expression of each gene. There is evidence to suggest that the RhoA protein has a half-life of up to 31 h [25], therefore a minimum gene knockdown period of 48 h was used to ensure an effect at the protein level. Negative controls treated with AllStars Negative Control siRNA (Qiagen, Hilden, Germany) were included in each experiment. All knockdown experiments were performed in triplicate. Knockdown of the *ARHGEF3* and *RHOA* genes did not appear to influence the proliferation or viability of any of the cell types studied.

### Knockdown in Osteoblast-like Cells

siRNA knockdown experiments were performed in 24-well tissue culture plates. For the Saos-2, hFOB 1.19 and MG-63 osteoblast-like cell lines, each well was seeded with 5×10^4^ cells. Cells were grown for 24 h before fresh medium was added to each culture and transfections were performed using a final siRNA concentration of 30 nM with 6 µL transfection reagent per well. Cells in each well were incubated with the transfection mix for 48 h at 37°C prior to washing with 1× PBS and extraction of total RNA.

### Knockdown in Peripheral Blood Mononuclear Cells/Osteoclast-like Cells

500 µL of freshly isolated PBMCs were aliquoted into 24-well tissue culture plates. Osteoclastogenesis was stimulated and confirmed microscopically and biochemically as described previously by TRAP staining. siRNA knockdown experiments were performed using a final siRNA concentration of 100 nM with 6 µL transfection reagent per well. Cells in each well were incubated with the transfection mix for 48 h at 37°C prior to washing with 1× PBS and extraction of total RNA.

### RNA Extraction and Microarray Analysis

A total of 18 RNA samples, 9 from Saos-2 and 9 from osteoclast-like cell cultures (donor 1) were used for the microarray analysis. Each set of 9 was comprised of 3 cultures treated with siRNA specific for *ARHGEF3*, 3 treated with siRNA specific for *RHOA* and 3 treated with negative control siRNA. Total RNA was extracted from each culture using the RNeasy Mini Kit (Qiagen, Hilden, Germany). The quality and quantity of all RNA samples was checked prior to microarray analysis using a 2100 Bioanalyzer (Agilent Technologies, Santa Clara, USA). 10 µL of each RNA sample was amplified using the TotalPrep RNA Amplification Kit (Applied Biosystems, Foster City, USA) before microarray analysis was performed using the HumanHT-12 v3 Expression BeadChip Kit (Illumina, San Diego, USA). The HumanHT-12 BeadChip profiles the expression of more than 25,000 annotated genes derived from NCBI RefSeq (Build 36.2) [26]. The complete results from the microarray analyses performed in this study have been submitted to The University of Western Australia’s Research Data Online resource.

### Gene Selection

While data were generated for most of the >25,000 genes included on the microarray, only 264 candidate genes were selected for statistical analysis in order to limit the potential for false positives. These candidate genes were selected on the basis of the following criteria: genes thought to have potentially important roles in osteoblast (n = 45) or osteoclast function (n = 62), or genes thought to play a role in the RhoA/ARHGEF3 signalling pathway (n = 157).

### Quantitative Real-time PCR

qRT-PCR was used to determine the degree of gene knockdown achieved and to validate microarray results for selected targets. Reverse transcription of RNA samples was first performed using the QuantiTect Reverse Transcription Kit (Qiagen, Hilden, Germany). The resulting cDNA was then amplified using the QuantiFast SYBR Green Kit (Qiagen, Hilden, Germany) in conjunction with an iQ5 Multicolor Real-Time PCR Detection System (Bio-Rad, Hercules, USA). cDNA samples were diluted in 1× TE buffer before analysis. QuantiTect Primer Assays (Qiagen, Hilden, Germany) were used to amplify most gene transcript sequences. Bioinformatics analysis revealed that the QuantiTect Primer Assay for the candidate gene *ACTA2* amplifies only one of the two transcript variants for this gene. Therefore, a custom primer pair was designed for this gene using the web-based Primer3 software package [27]. The human 18S ribosomal RNA gene (*RRN18S*) was selected as an internal reference for this work to allow for normalisation of the data for variations in the quantity of cDNA added to each reaction. The reaction efficiency of each primer pair was calculated by amplifying a 10-fold dilution series of target sequence across 5 orders of magnitude. This was performed to confirm that the amplification efficiency of each gene of interest is no more than 10% from that of the internal reference as recommended by Schmittgen and Livak [28]. The log template dilution (x-axis) was plotted against the cycle threshold (C_T_) value obtained for each dilution (y-axis) with the slope of the line used for calculation of amplification efficiency using the equation *m* = −(1/log *E*), where *m* is the slope of the line and *E* is the reaction efficiency. A reaction efficiency of 2.0 equates to a perfect doubling of amplicon product during each PCR cycle. All reactions were performed in triplicate with the mean C_T_ value used in the statistical analysis. Melting-curve analysis was performed on all real-time PCR products to confirm amplification of a single DNA sequence. A random selection of PCR products were also subjected to agarose gel electrophoresis for additional confirmation of the specificity of amplification.

### Microarray Statistical Analysis

Differential expression analysis of the microarray data using the Illumina custom error model was performed using the BeadStudio v3.4.0 software package (Illumina, San Diego, USA). Samples treated with the negative control siRNA were specified as the reference group. The raw microarray gene expression data were normalised using the quantile normalisation algorithm [29], which adjusts the sample signals to minimise the influence of variation arising from non-biological factors (eg. pipetting variation) [30]. Background subtraction was performed on the data to minimise the variation in background noise between arrays and to remove signal resulting from non-specific hybridisation [31]. Once background subtraction has been performed on the data, the expected signal for unexpressed targets is zero. The data were corrected for multiple testing using the Benjamini-Hochberg False Discovery Rate algorithm [32].

### Real-time PCR Statistical Analysis

Gene expression ratios were calculated using the comparative C_T_ method as described by Schmittgen and Livak [28]. Briefly, the ΔC_T_ (C_T_ of the test gene−C_T_ of the internal reference) was calculated for each gene of interest in each sample in the test and control groups. This figure was then entered into the equation 2^−ΔC^_T_ with the mean ± standard error calculated for each of the test and control groups. 2^−ΔC^_T_ values for test and control groups were analysed using an unpaired t-test to determine whether differences in expression were statistically significant. Combined 2^−ΔC^_T_ values for the osteoclast-like cells were examined by 2-way analysis of variance (ANOVA) (note that this combined analysis was not performed for the osteoblast-like cells due to potential variation in the maturation state and gene expression profile of each cell line). Significant associations are defined as *P<0.05*.

## Results

### Osteoblast Microarray Results

**Figure 1:**
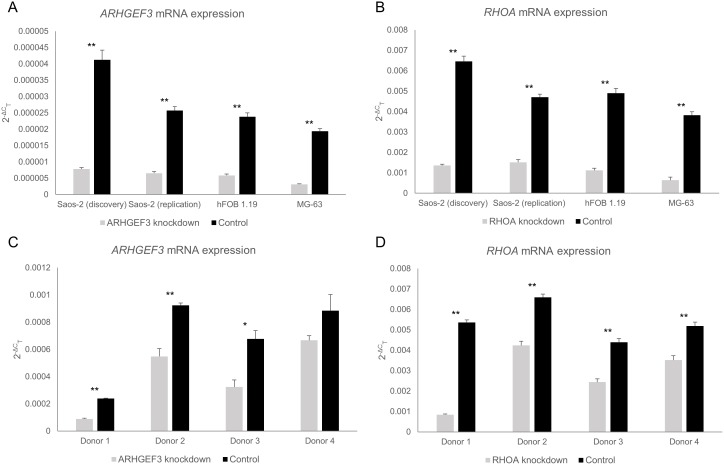
qRT-PCR validation of *ARHGEF3* and *RHOA* gene knockdown in each cell type. (A) *ARHGEF3* mRNA expression in osteoblast-like cells. (B) *RHOA* mRNA expression in osteoblast-like cells. (C) *ARHGEF3* mRNA expression in osteoclast-like cells. (D) *RHOA* mRNA expression in osteoclast-like cells. Data displayed as mean 2^−ΔC^_T_ ± SEM from three biological replicates. *P<0.05, **P<0.01.

Knockdown of the *ARHGEF3* and *RHOA* genes was validated in the Saos-2 cells by qRT-PCR prior to microarray analysis. For the *ARHGEF3* and *RHOA* genes, 81% and 79% knockdown was achieved respectively in these cells (Fig. 1A and B). Of the 202 candidate genes examined in the osteoblast-like cells, gene knockdown resulted in significant changes in expression of 10 genes after adjustment for multiple testing (Table 1). Knockdown of *ARHGEF3* resulted in significant changes to the expression of 8 genes: *TNFRSF11B*, *SP7*, *ALPL*, *ANGPTL2*, *GNA11*, *MYO9B*, *GNAI2* and *PFN1*. For *RHOA* knockdown, 2 genes were affected: *PTH1R* and *ACTA2*. Table S1 contains the microarray results for all of the candidate genes examined in the Saos-2 cells (*P* values corrected for multiple testing).

**Table 1:** Genes significantly influenced in microarray analysis of *ARHGEF3* and *RHOA* gene knockdown in Saos-2 osteoblast-like cells.

Knockdown	Gene	Geneproduct	Meanknockdown*FU	Meancontrol*FU	Expressionratio	*P*
*ARHGEF3*	*TNFRSF11B*	Osteoprotegerin	235	308	0.76	<0.001
	*SP7*	Osterix	434	381	1.14	0.007
	*ALPL*	Alkalinephosphatase	19262	17173	1.12	0.03
	*ANGPTL2*	Angiopoietin-like 2	81	50	1.64	<0.001
	*GNA11*	Guanine nucleotide bindingprotein alpha 11	352	469	0.75	0.002
	*MYO9B*	Myosin IXB	165	212	0.78	0.005
	*GNAI2*	Guanine nucleotide bindingprotein alpha inhibiting activity polypeptide 2	1106	1330	0.83	0.006
	*PFN1*	Profilin 1	7458	8220	0.91	0.02
*RHOA*	*PTH1R*	Parathyroid hormone 1 receptor	1166	492	2.37	0.002
	*ACTA2*	Alpha 2 actin, smooth muscle	3059	7812	0.39	<0.001

Expression ratios are given as expression of the gene in the knockdown cultures relative to the negative control cultures. *P* adjusted for multiple testing.

*FU, fluorescence units.

### qRT-PCR Validation and Replication of Microarray Results for Targeted Genes in Osteoblast-like Cell Lines

Both of the differentially regulated genes in the *RHOA* knockdown group (*PTH1R* and *ACTA2*) and 2 from the *ARHGEF3* knockdown group (*TNFRSF11B* and *ALPL*) were then selected for confirmatory and replication studies using qRT-PCR. While the microarray results suggested that 8 of the 202 genes examined could potentially be regulated by *ARHGEF3*, the *TNFRSF11B* and *ALPL* genes were selected based on a number of factors including their importance to bone metabolism, their level of expression in the cell type and the size and statistical significance of the regulatory effect. These 4 genes were thus examined in one additional replication study experiment in Saos-2 cells as well as in two additional osteoblast-like cell lines, hFOB 1.19 and MG-63. For the *ARHGEF3* and *RHOA* genes, 75% and 68% knockdown was achieved respectively in the replication batch of Saos-2 cells, 75% and 77% respectively in the hFOB 1.19 cells and 84% and 83% respectively in the MG-63 cells (Fig. 1A and B). The average knockdown achieved across all of the osteoblast-like cell lines as determined by qRT-PCR was 76.8% for *RHOA* and 78.7% for *ARHGEF3* (Fig. 1A and B).

**Figure 2:**
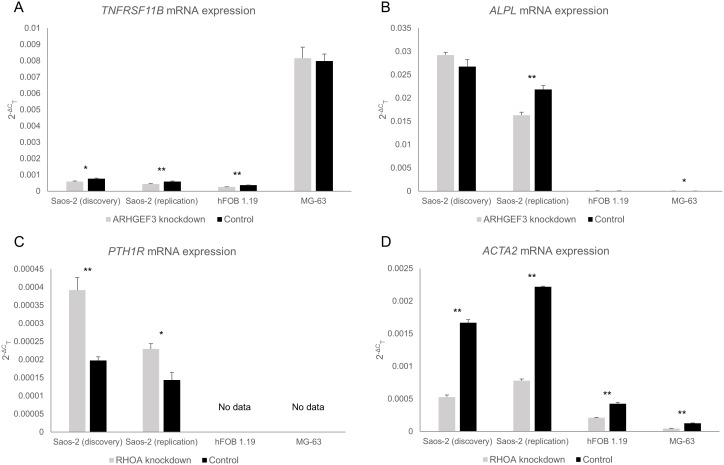
qRT-PCR validation of selected genes in response to *ARHGEF3* and *RHOA* gene knockdown in osteoblast-like cells. (A) *TNFRSF11B* mRNA expression in osteoblast-like cells. (B) *ALPL* mRNA expression in osteoblast-like cells. (C) *PTH1R* mRNA expression in osteoblast-like cells. (D) *ACTA2* mRNA expression in osteoblast-like cells. Data displayed as mean 2^−ΔC^_T_ ± SEM from three biological replicates. *P<0.05, **P<0.01.

The influence of gene knockdown on *TNFRSF11B*, *ALPL*, *PTH1R* and *ACTA2* expression is shown in Fig. 2A–D. A highly significant down regulation of *ACTA2* was observed in response to *RHOA* knockdown in each of the osteoblast-like cell lines examined (*P<0.001*). The qRT-PCR results also confirmed the up-regulation of the *PTH1R* gene in response to *RHOA* knockdown observed in the microarray screen (*P<0.001*); however, neither the hFOB 1.19 nor the MG-63 cell lines expressed this particular gene. The qRT-PCR studies showed *ARHGEF3* knockdown had a significant influence on *TNFRSF11B* expression in the Saos-2 and hFOB 1.19 cell lines (*P = 0.003–0.02*), however little influence was seen in the MG-63 cells. *ARHGEF3* knockdown had no consistent influence on *ALPL* expression.

### Osteoclast Microarray Results

The osteoclastic phenotype of the cells was confirmed by expression of the genes encoding the osteoclastic biochemical markers TRAP (*ACP5*), cathepsin K (*CTSK*) and calcitonin receptor (*CALCR*) from the microarray output. The *ACP5* and *CTSK* genes were found to be expressed at particularly high levels in this cell type (mean microarray signal >14,000 fluorescence units).

Knockdown of the *ARHGEF3* and *RHOA* genes was validated in the osteoclast-like cells by qRT-PCR prior to microarray analysis. For the *ARHGEF3* and *RHOA* genes, a mean knockdown of 63% and 84% was achieved respectively in the osteoclast-like cells from donor 1 (Fig. 1C and D). Of the 219 candidate genes examined in this cell type, gene knockdown resulted in significant changes in expression of 17 genes after adjustment for multiple testing (Table 2). *ARHGEF3* knockdown was found to significantly influence the expression of 12 genes: *CCL5*, *HLA-C*, *SNCA*, *TNF*, *OSCAR*, *CD44*, *BIRC3*, *ITGB7*, *ITGAE*, *ITGAL*, *ITGA3* and *ITGAM*. For *RHOA* knockdown, 9 genes were found to be significantly influenced: *TNF*, *THBS2*, *CCL5*, *ITGB7*, *ARHGDIA*, *IGF1*, *ACTA2*, *MYL9* and *ITGAE*. Of these, the effect of *RHOA* knockdown on the *ACTA2* gene was also observed in the osteoblast-like cells. Table S2 contains the microarray results for all of the candidate genes examined in the osteoclast-like cells (*P* values corrected for multiple testing).

**Table 2:** Genes significantly influenced in microarray analysis of *ARHGEF3* and *RHOA* gene knockdown in osteoclast-like cells from donor 1.

Knockdown	Gene	Gene product	Meanknockdown*FU	Meancontrol*FU	Expressionratio	*P*
*ARHGEF3*	*CCL5*	Chemokine ligand 5	4402	588	7.48	<0.001
	*HLA-C*	Major histocompatibility complex,class I, C	235	87	2.71	<0.001
	*SNCA*	Synuclein, alpha	129	260	0.49	<0.001
	*TNF*	Tumour necrosisfactor alpha	420	156	2.69	<0.001
	*OSCAR*	Osteoclast associatedIg-like receptor	1950	1244	1.57	0.01
	*CD44*	CD44 molecule	2279	3360	0.68	0.03
	*BIRC3*	Baculoviral IAP repeat-containing 3	496	321	1.55	0.04
	*ITGB7*	Integrin, beta 7	588	106	5.54	<0.001
	*ITGAE*	Integrin, alpha E	256	483	0.53	<0.001
	*ITGAL*	Integrin, alpha L	145	79	1.85	0.003
	*ITGA3*	Integrin, alpha 3	107	221	0.48	0.003
	*ITGAM*	Integrin, alpha M	1033	1699	0.61	0.004
*RHOA*	*TNF*	Tumour necrosisfactor alpha	345	156	2.21	<0.001
	*THBS2*	Thrombospondin 2	89	43	2.05	<0.001
	*CCL5*	Chemokine ligand 5	891	588	1.52	0.006
	*ITGB7*	Integrin, beta 7	246	106	2.32	<0.001
	*ARHGDIA*	Rho GDP dissociationinhibitor alpha	579	998	0.58	<0.001
	*IGF1*	Insulin-like growth factor 1	121	42	2.86	<0.001
	*ACTA2*	Alpha 2 actin, smooth muscle	545	965	0.56	0.001
	*MYL9*	Myosin, light chain 9, regulatory	22	49	0.46	0.04
	*ITGAE*	Integrin, alpha E	342	483	0.71	0.04

Expression ratios are given as expression of the gene in the knockdown cultures relative to the negative control cultures. *P* adjusted for multiple testing.

*FU, fluorescence units.

### qRT-PCR Validation and Replication of Microarray Results for Targeted Genes in Osteoclast-like Cells

**Figure 3:**
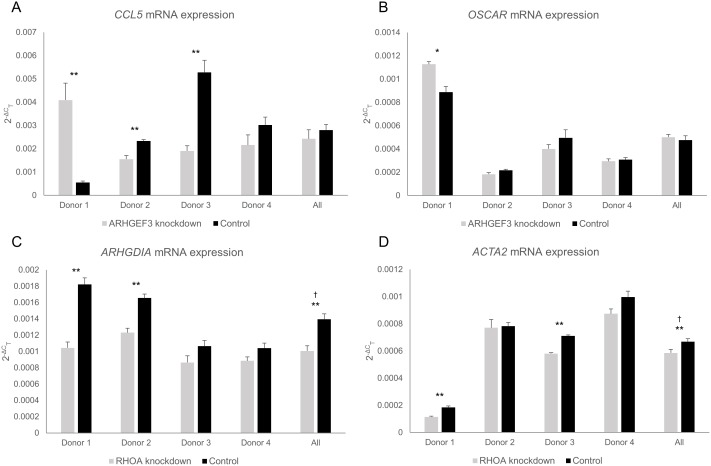
qRT-PCR validation of selected genes in response to *ARHGEF3* and *RHOA* gene knockdown in osteoclast-like cells. (A) *CCL5* mRNA expression in osteoclast-like cells. (B) *OSCAR* mRNA expression in osteoclast-like cells. (C) *ARHGDIA* mRNA expression in osteoclast-like cells. (D) *ACTA2* mRNA expression in osteoclast-like cells. Data displayed as mean 2^−ΔC^_T_ ± SEM from three biological replicates. *P<0.05, **P<0.01, † determined as significant by ANOVA.

In the osteoclast studies, two of the differentially regulated genes from each of the knockdown experiments were selected for validation and replication analysis by qRT-PCR in osteoclast-like cells from 3 additional donors. These included the *CCL5* and *OSCAR* genes for *ARHGEF3* knockdown, and *ARHGDIA* and *ACTA2* genes for *RHOA* knockdown (Fig. 3A–D).

For the *ARHGEF3* and *RHOA* genes, 41% and 36% knockdown was achieved respectively in the donor 2 cells, 52% and 45% respectively in the donor 3 cells and 25% and 32% respectively in the donor 4 cells (Fig. 1C and D). The efficiency of knockdown of *ARHGEF3* and *RHOA* averaged 45.3% and 49.3% respectively in this cell type, substantially lower than that observed in the osteoblast-like cells (76.8% vs 49.3% for *RHOA*, *P = 0.07*; 78.7% vs 45.3% for *ARHGEF3*, *P = 0.007*). The knockdown was considerably lower than desired, however there was some evidence from the overall analysis to suggest that knockdown of *RHOA* reduces the expression of *ACTA2* in this cell type (*P = 0.002* by ANOVA). *RHOA* knockdown also caused a significant overall reduction in *ARHGDIA* expression in the osteoclast-like cells (*P<0.001* by ANOVA). While some significant changes were seen for cells from particular donors, the influence of *ARHGEF3* knockdown on *CCL5* and *OSCAR* was inconsistent.

## Discussion

We previously reported associations between polymorphism in the *RHOA* and *ARHGEF3* genes and bone density in women and in this study investigated the potential role of these genes in the regulation of bone cells. The knockdown of these two genes showed clear effects on the expression of a number of potentially relevant genes and pathways in two of the major bone cell types – osteoblasts and osteoclasts. Greater gene knockdown levels were achieved in the osteoblast-like cells than in the osteoclast-like cells.

Concerning the studies performed in the osteoblast-like cells, expression of the *ACTA2* gene was found to be significantly down-regulated by *RHOA* knockdown in all three osteoblast-like cell lines examined (Saos-2, hFOB 1.19 and MG-63), with an average expression ratio of 0.35 seen in knockdown cell cultures relative to control cell cultures by qRT-PCR. The *ACTA2* gene encodes the alpha 2 actin cytoskeletal protein, which is a major component of the smooth muscle cell contractile apparatus and accounts for around 40% of the total protein and around 70% of the total actin in smooth muscle cells [33], [34]. There have been few studies on the role of the *ACTA2* gene product in bone metabolism, however there is evidence in the literature to suggest that the *ACTA2* gene is regulated by RhoA signalling. Mack *et al.* [35] found that expression of constitutively active RhoA in rat smooth muscle cell cultures increased the activity of the *Acta2* promoter, whereas inhibition of RhoA decreased the activity of the promoter. They also found that stimulation of actin polymerisation in these smooth muscle cells increased the activity of the *Acta2* promoter by 13-fold [35]. In addition, Zhao *et al.* [36] reported that static tensile forces applied to rat fibroblasts stimulates the promoter activity of the *Acta2* gene through the Rho signalling pathway. Collectively, these data suggest that expression of the *ACTA2* gene may be regulated through the RhoA signalling pathway, and the results presented here support this.

Knockdown of the *ARHGEF3* gene in both the discovery and replication experiments with Saos-2 cells resulted in significant down-regulation of the levels of *TNFRSF11B* (osteoprotegerin) mRNA. This effect was replicated in the hFOB 1.19 cells, but not in the MG-63 cell line. It is not clear why this effect was not seen in the MG-63 cells, it may be an effect specific to that cell line. *TNFRSF11B* mRNA levels were significantly higher in the MG-63 cells than in the hFOB 1.19 and Saos-2 cells, in line with studies by Pautke et al. [37]. There are well described differences in the expression patterns between osteoblast-like cell lines reported elsewhere [37]–[39].

In addition to these findings, knockdown of the *RHOA* gene in both the discovery and replication batches of Saos-2 cells resulted in significant up-regulation of *PTH1R* (parathyroid hormone 1 receptor) mRNA levels, although expression of this gene was not detected in the hFOB 1.19 or MG-63 cell lines. Both *PTH1R* and *TNFRSF11B* have a major role in the stimulation of osteoclastogenesis upon exposure to parathyroid hormone (PTH), suggesting that the *ARHGEF3* and *RHOA* genes may be involved in this process. Radeff *et al.* [40] found that treatment of UMR-106 rat osteoblast-like cells with Clostridium difficile toxin B, which specifically inhibits the Rho proteins (including RhoA) through glucosylation of the nucleotide binding site [41], reduced PTH-induced expression of the *Il6* gene, the product of which has been shown to promote osteoclastogenesis [42]. The authors concluded that the Rho proteins are an important component of PTH signalling in osteoblasts and may have a role in the activation of the intracellular messenger protein kinase C alpha [40]. Another study published by Wang and Stern [43] found that UMR-106 rat osteoblast-like cells transfected with dominant negative RhoA and treated with PTH and/or calcitriol increased production of *TNFSF11* mRNA (encoding RANKL) and reduced production of *TNFRSF11B* mRNA, stimulating osteoclastogenesis of co-cultured RAW 264.7 mouse monocyte/macrophage-like cells [43]. However, when these cells were transfected with constitutively active RhoA and treated with PTH and/or calcitriol, the levels of *TNFSF11* and *TNFRSF11B* mRNA did not change significantly and osteoclastogenesis of the RAW 264.7 cells failed to occur [43]. These results led the authors to suggest that RhoA signalling can inhibit hormone-stimulated osteoclastogenesis through effects on RANKL and osteoprotegerin expression in osteoblasts [43]. No consistent effect of *ARHGEF3* knockdown on *ALPL* expression could be found, however a higher expression of this gene in Saos-2 cells was found compared to the other cells investigated, including the MG-63 cells, in line with the findings of Pautke et al. [37].

One limitation of the gene expression data in the osteoclast-like cells was that consistently high gene knockdown (>60%) was not achieved in some of our experiments, and a greater level of knockdown may show more substantial changes than seen in our studies. Nevertheless, some interesting results were obtained. Expression of the *ARHGDIA* and *ACTA2* genes was found to be significantly reduced in response to *RHOA* gene knockdown. The product of the *ARHGDIA* gene is a Rho GDP dissociation inhibitor (GDI) which acts as a negative regulator of several of the RhoGTPases [44]. RhoGDIs maintain the Rho proteins in their inactive GDP-bound state by inhibiting the exchange of GDP for GTP [45] and by restricting membrane anchoring [46]. The down-regulation of *ARHGDIA* expression seen in the *RHOA* knockdown osteoclast-like cells in this study could be a compensatory mechanism for the reduced expression of the *RHOA* gene. The influence of *RHOA* knockdown on expression of the *ACTA2* gene adds further support to the earlier suggestion that expression of this gene is regulated by the RhoA signalling pathway.

In conclusion, knockdown of the *ARHGEF3* and *RHOA* genes in bone cells of human origin reveals important regulatory changes including significant down-regulation of the *ACTA2* gene, encoding the cytoskeletal protein alpha 2 actin, in both osteoblast-like and osteoclast-like cells in response to *RHOA* knockdown. *RHOA* knockdown also resulted in up-regulation of the *PTH1R* gene in the Saos-2 osteoblast-like cell line and down-regulation of *ARHGDIA* in osteoclast-like cells, whereas *ARHGEF3* knockdown caused down-regulation of the *TNFRSF11B* gene in the Saos-2 and hFOB 1.19 osteoblast-like cells. These findings add further evidence to previous studies suggesting a role for the *RHOA* and *ARHGEF3* genes in bone metabolism. Future work in this area could include confirmatory studies investigating the influence of over-expression of the *ARHGEF3* and *RHOA* genes in these cell types and examination of effects at the protein level.

## Supporting Information

Table S1 **Microarray results including** ***P*** **values adjusted for multiple-testing for all of the candidate genes examined in the Saos-2 cells.**
(XLSX)

Table S2 **Microarray results including** ***P*** **values adjusted for multiple-testing for all of the candidate genes examined in the osteoclast-like cells.**
(XLSX)

## References

[1] Kanis JA, Melton LJ 3rd, Christiansen C, Johnston CC, Khaltaev N (1994) The diagnosis of osteoporosis. J Bone Miner Res 9: 1137–1141. 7976495 10.1002/jbmr.5650090802

[2] Prince RL, Dick I (1997) Oestrogen effects on calcium membrane transport: a new view of the inter-relationship between oestrogen deficiency and age-related osteoporosis. Osteoporos Int 7 Suppl 3 S150–154. 9536322 10.1007/BF03194362

[3] Flicker L, Hopper JL, Rodgers L, Kaymakci B, Green RM, et al (1995) Bone density determinants in elderly women: a twin study. J Bone Miner Res 10: 1607–1613. 8592936 10.1002/jbmr.5650101102

[4] Michaelsson K, Melhus H, Ferm H, Ahlbom A, Pedersen NL (2005) Genetic liability to fractures in the elderly. Arch Intern Med 165: 1825–1830. 16157825 10.1001/archinte.165.16.1825

[5] Evans RA, Marel GM, Lancaster EK, Kos S, Evans M, et al (1988) Bone mass is low in relatives of osteoporotic patients. Ann Intern Med 109: 870–873. 3190041 10.7326/0003-4819-109-11-870

[6] Pocock NA, Eisman JA, Hopper JL, Yeates MG, Sambrook PN, et al (1987) Genetic determinants of bone mass in adults. A twin study. J Clin Invest 80: 706–710. 3624485 10.1172/JCI113125 PMC442294

[7] Seeman E, Hopper JL, Young NR, Formica C, Goss P, et al (1996) Do genetic factors explain associations between muscle strength, lean mass, and bone density? A twin study. Am J Physiol 270: E320–327. 8779955 10.1152/ajpendo.1996.270.2.E320

[8] Andrew T, Antioniades L, Scurrah KJ, Macgregor AJ, Spector TD (2005) Risk of wrist fracture in women is heritable and is influenced by genes that are largely independent of those influencing BMD. J Bone Miner Res 20: 67–74. 15619671 10.1359/JBMR.041015

[9] Deng HW, Chen WM, Recker S, Stegman MR, Li JL, et al (2000) Genetic determination of Colles’ fracture and differential bone mass in women with and without Colles’ fracture. J Bone Miner Res 15: 1243–1252. 10893672 10.1359/jbmr.2000.15.7.1243

[10] Ioannidis JP, Ng MY, Sham PC, Zintzaras E, Lewis CM, et al (2007) Meta-analysis of genome-wide scans provides evidence for sex- and site-specific regulation of bone mass. J Bone Miner Res 22: 173–183. 17228994 10.1359/jbmr.060806 PMC4016811

[11] Lee YH, Rho YH, Choi SJ, Ji JD, Song GG (2006) Meta-analysis of genome-wide linkage studies for bone mineral density. J Hum Genet 51: 480–486. 16534542 10.1007/s10038-006-0390-9

[12] Xiao P, Shen H, Guo YF, Xiong DH, Liu YZ, et al (2006) Genomic regions identified for BMD in a large sample including epistatic interactions and gender-specific effects. J Bone Miner Res 21: 1536–1544. 16995807 10.1359/jbmr.060717

[13] Wilson SG, Reed PW, Bansal A, Chiano M, Lindersson M, et al (2003) Comparison of genome screens for two independent cohorts provides replication of suggestive linkage of bone mineral density to 3p21 and 1p36. Am J Hum Genet 72: 144–155. 12478480 10.1086/345819 PMC378619

[14] Wynne F, Drummond FJ, Daly M, Brown M, Shanahan F, et al (2003) Suggestive linkage of 2p22-25 and 11q12-13 with low bone mineral density at the lumbar spine in the Irish population. Calcif Tissue Int 72: 651–658. 14562992 10.1007/s00223-002-2086-2

[15] Mullin BH, Prince RL, Dick IM, Hart DJ, Spector TD, et al (2008) Identification of a role for the ARHGEF3 gene in postmenopausal osteoporosis. Am J Hum Genet 82: 1262–1269. 18499081 10.1016/j.ajhg.2008.04.016 PMC2427258

[16] Mullin BH, Prince RL, Mamotte C, Spector TD, Hart DJ, et al (2009) Further genetic evidence suggesting a role for the RhoGTPase-RhoGEF pathway in osteoporosis. Bone 45: 387–391. 19427924 10.1016/j.bone.2009.04.254

[17] Arthur WT, Ellerbroek SM, Der CJ, Burridge K, Wennerberg K (2002) XPLN, a guanine nucleotide exchange factor for RhoA and RhoB, but not RhoC. J Biol Chem 277: 42964–42972. 12221096 10.1074/jbc.M207401200

[18] Etienne-Manneville S, Hall A (2002) Rho GTPases in cell biology. Nature 420: 629–635. 12478284 10.1038/nature01148

[19] McBeath R, Pirone DM, Nelson CM, Bhadriraju K, Chen CS (2004) Cell shape, cytoskeletal tension, and RhoA regulate stem cell lineage commitment. Dev Cell 6: 483–495. 15068789 10.1016/s1534-5807(04)00075-9

[20] Meyers VE, Zayzafoon M, Douglas JT, McDonald JM (2005) RhoA and cytoskeletal disruption mediate reduced osteoblastogenesis and enhanced adipogenesis of human mesenchymal stem cells in modeled microgravity. J Bone Miner Res 20: 1858–1866. 16160744 10.1359/JBMR.050611 PMC1351020

[21] Chellaiah MA, Soga N, Swanson S, McAllister S, Alvarez U, et al (2000) Rho-A is critical for osteoclast podosome organization, motility, and bone resorption. J Biol Chem 275: 11993–12002. 10766830 10.1074/jbc.275.16.11993

[22] Fogh J, Fogh JM, Orfeo T (1977) One hundred and twenty-seven cultured human tumor cell lines producing tumors in nude mice. J Natl Cancer Inst 59: 221–226. 327080 10.1093/jnci/59.1.221

[23] Harris SA, Enger RJ, Riggs BL, Spelsberg TC (1995) Development and characterization of a conditionally immortalized human fetal osteoblastic cell line. J Bone Miner Res 10: 178–186. 7754797 10.1002/jbmr.5650100203

[24] Billiau A, Edy VG, Heremans H, Van Damme J, Desmyter J, et al (1977) Human interferon: mass production in a newly established cell line, MG-63. Antimicrob Agents Chemother 12: 11–15. 883813 10.1128/aac.12.1.11 PMC352146

[25] Backlund PS Jr (1997) Post-translational processing of RhoA. Carboxyl methylation of the carboxyl-terminal prenylcysteine increases the half-life of Rhoa. J Biol Chem 272: 33175–33180. 9407105 10.1074/jbc.272.52.33175

[26] Illumina (2008) HumanHT-12 v3 Expression BeadChip. Illumina Technical Bulletin Pub. No.470-2008-005.

[27] Rozen S, Skaletsky H (2000) Primer3 on the WWW for general users and for biologist programmers. Methods Mol Biol 132: 365–386. 10547847 10.1385/1-59259-192-2:365

[28] Schmittgen TD, Livak KJ (2008) Analyzing real-time PCR data by the comparative C(T) method. Nat Protoc 3: 1101–1108. 18546601 10.1038/nprot.2008.73

[29] Bolstad BM, Irizarry RA, Astrand M, Speed TP (2003) A comparison of normalization methods for high density oligonucleotide array data based on variance and bias. Bioinformatics 19: 185–193. 12538238 10.1093/bioinformatics/19.2.185

[30] Illumina (2007) BeadStudio Normalization Algorithms for Gene Expression Data. Illumina Technical Bulletin Pub. No.470-2007-005.

[31] Dunning MJ, Barbosa-Morais NL, Lynch AG, Tavare S, Ritchie ME (2008) Statistical issues in the analysis of Illumina data. BMC Bioinformatics 9: 85. 18254947 10.1186/1471-2105-9-85 PMC2291044

[32] Benjamini Y, Hochberg Y (1995) Controlling the False Discovery Rate: a Practical and Powerful Approach to Multiple Testing. Journal of the Royal Statistical Society: Series B 57: 289–300.

[33] Fatigati V, Murphy RA (1984) Actin and tropomyosin variants in smooth muscles. Dependence on tissue type. J Biol Chem 259: 14383–14388. 6501298

[34] Owens GK (1995) Regulation of differentiation of vascular smooth muscle cells. Physiol Rev 75: 487–517. 7624392 10.1152/physrev.1995.75.3.487

[35] Mack CP, Somlyo AV, Hautmann M, Somlyo AP, Owens GK (2001) Smooth muscle differentiation marker gene expression is regulated by RhoA-mediated actin polymerization. J Biol Chem 276: 341–347. 11035001 10.1074/jbc.M005505200

[36] Zhao XH, Laschinger C, Arora P, Szaszi K, Kapus A, et al (2007) Force activates smooth muscle alpha-actin promoter activity through the Rho signaling pathway. J Cell Sci 120: 1801–1809. 17456553 10.1242/jcs.001586

[37] Pautke C, Schieker M, Tischer T, Kolk A, Neth P, et al (2004) Characterization of osteosarcoma cell lines MG-63, Saos-2 and U-2 OS in comparison to human osteoblasts. Anticancer Res 24: 3743–3748. 15736406

[38] Bilbe G, Roberts E, Birch M, Evans DB (1996) PCR phenotyping of cytokines, growth factors and their receptors and bone matrix proteins in human osteoblast-like cell lines. Bone 19: 437–445. 8922641 10.1016/s8756-3282(96)00254-2

[39] Subramaniam M, Jalal SM, Rickard DJ, Harris SA, Bolander ME, et al (2002) Further characterization of human fetal osteoblastic hFOB 1.19 and hFOB/ER alpha cells: bone formation in vivo and karyotype analysis using multicolor fluorescent in situ hybridization. J Cell Biochem 87: 9–15. 12210717 10.1002/jcb.10259

[40] Radeff JM, Nagy Z, Stern PH (2004) Rho and Rho kinase are involved in parathyroid hormone-stimulated protein kinase C alpha translocation and IL-6 promoter activity in osteoblastic cells. J Bone Miner Res 19: 1882–1891. 15476589 10.1359/JBMR.040806

[41] Wilkins TD, Lyerly DM (1996) Clostridium difficile toxins attack Rho. Trends Microbiol 4: 49–51. 8820565 10.1016/0966-842X(96)81508-3

[42] Lowik CW, van der Pluijm G, Bloys H, Hoekman K, Bijvoet OL, et al (1989) Parathyroid hormone (PTH) and PTH-like protein (PLP) stimulate interleukin-6 production by osteogenic cells: a possible role of interleukin-6 in osteoclastogenesis. Biochem Biophys Res Commun 162: 1546–1552. 2548501 10.1016/0006-291x(89)90851-6

[43] Wang J, Stern PH (2010) Osteoclastogenic activity and RANKL expression are inhibited in osteoblastic cells expressing constitutively active Galpha(12) or constitutively active RhoA. J Cell Biochem. 10.1002/jcb.22883 PMC3478942 20872746

[44] DerMardirossian C, Bokoch GM (2005) GDIs: central regulatory molecules in Rho GTPase activation. Trends Cell Biol 15: 356–363. 15921909 10.1016/j.tcb.2005.05.001

[45] Fukumoto Y, Kaibuchi K, Hori Y, Fujioka H, Araki S, et al (1990) Molecular cloning and characterization of a novel type of regulatory protein (GDI) for the rho proteins, ras p21-like small GTP-binding proteins. Oncogene 5: 1321–1328. 2120668

[46] Olofsson B (1999) Rho guanine dissociation inhibitors: pivotal molecules in cellular signalling. Cell Signal 11: 545–554. 10433515 10.1016/s0898-6568(98)00063-1

